# Establishment and evaluation of predictive model for acute heart failure after PCI in patients with STEMI

**DOI:** 10.3389/fcvm.2026.1648797

**Published:** 2026-04-14

**Authors:** Jun Li, Fachao Shi, Damin Huang, Dongmei Yue

**Affiliations:** 1 Department of Cardiology, Chongming Hospital Affiliated to Shanghai University of Medicine and Health Sciences, Shanghai, China; 2 Department of Cardiology, Ma’anshan People’s Hospital, Ma'anshan Hospital Affiliated to Wannan Medical College, Anhui, China

**Keywords:** acute heart failure, AHF, PCI, predictive model, ST-segment elevation myocardial infarction

## Abstract

**Objective:**

To establish a predictive model for acute heart failure (AHF) occurrence in patients with ST-segment elevation myocardial infarction (STEMI) following percutaneous coronary intervention (PCI) and evaluate its clinical performance.

**Methods:**

A retrospective analysis was conducted on 419 STEMI patients treated at the Cardiology Department of Maanshan People's Hospital from January 2018 to December 2024. Patients were divided into AHF group (*n* = 100) and non-AHF group (*n* = 319) based on AHF occurrence. Logistic regression analysis identified independent risk factors for model construction. Model performance was assessed using receiver operating characteristic (ROC) curves with optimal threshold determination via maximum Youden index. Statistical validation included Omnibus and Hosmer-Lemeshow tests.

**Results:**

The AHF prediction model was: Logit(P) = 3.084 − 0.026 × systolic blood pressure + 0.083 × neutrophil count − 0.041 × total bilirubin + 0.238 × urea nitrogen − 0.045 × left ventricular ejection fraction (LVEF). AHF was predicted when logit(P) > 0.231. Statistical validation showed Omnibus test *χ*^2^ = 7.112, *P* = 0.008, and Hosmer-Lemeshow test *χ*^2^ = 6.551, *P* = 0.586, indicating good model fit. The model achieved 74% sensitivity, 86.8% specificity, and 82.4% diagnostic accuracy. Comparative ROC analysis demonstrated superior performance vs. established scores: predictive model + Grace Score 0.902 vs. baseline model 0.715 (Delong test: Z = 0.235, *P* < 0.0001), with continuous net reclassification index (NRI) 0.7472 (*P* < 0.0001) and integrated discrimination index (IDI) 0.2455 (*P* < 0.0001); predictive model + CAMI-STEMI Score 0.909 vs. baseline model 0.717 (Delong test: *Z* = 0.4930, *P* < 0.0001), with continuous NRI 0.7245 (*P* < 0.0001) and IDI 0.2101 (*P* < 0.0001). Clinical decision curve analysis demonstrated net clinical benefit when threshold probability ranged from 0.1 to 0.99.

**Conclusion:**

Systolic blood pressure, total bilirubin, LVEF, neutrophils, and urea nitrogen are independent factors affecting AHF occurrence after PCI in STEMI patients. This exploratory predictive model demonstrates potential for assessing AHF risk in STEMI patients following PCI and may provide valuable clinical decision support for risk stratification and patient management. However, external validation in independent, multicenter populations is essential before clinical implementation.

## 1 Introduction

In patients with ST-segment elevation myocardial infarction (STEMI), although advancements in percutaneous coronary intervention (PCI) techniques have significantly improved patient survival rates (1), the occurrence of postoperative acute heart failure (AHF) remains one of the important factors affecting long-term prognosis (2). The development of AHF is closely related to myocardial ischemia, ventricular remodeling, and heart failure, which not only increases the risk of mortality during hospitalization but also has a profound impact on the patients’ quality of life (3, 4). Therefore, establishing and evaluating predictive models for acute heart failure is of great significance for clinical practice.

Firstly, accurately identifying high-risk patients can help clinicians implement more proactive monitoring and intervention measures after PCI, thereby reducing the incidence of AHF. Studies have shown that female patients often have poorer presentations and prognoses in acute myocardial infarction, and the proportion of females receiving reperfusion therapy is lower than that of males, further emphasizing the need for attention to high-risk populations (5, 6). Secondly, the application of predictive models can optimize resource allocation, reduce unnecessary medical expenditures, and improve the efficiency of healthcare services (7). Additionally, with a deeper understanding of the pathophysiological mechanisms of heart failure, integrated assessment methods based on clinical data and biomarkers are expected to provide a basis for individualized treatment, thereby improving patient outcomes (8–10).

In the current research context, predictive models for acute heart failure not only provide a scientific basis for clinical decision-making but also indicate future research directions. By analyzing different clinical characteristics and biomarkers, researchers can better understand the mechanisms underlying the occurrence of AHF, thus promoting the development and application of new therapies (11, 12). Therefore, the establishment and evaluation of predictive models for AHF following PCI in patients with STEMI holds significant clinical importance and necessity.

## 2 Materials and methods

### 2.1 Study population

A retrospective analysis was conducted on 419 STEMI patients who underwent emergency PCI at Maanshan People's Hospital from January 2018 to December 2024. All participants were patients who received stent implantation. AHF events were assessed prospectively during the first 7 days (168 h) following PCI, based on guideline-recommended definitions. Patients who died within 7 days post-PCI without documented evidence of acute heart failure were excluded from the final analysis, due to unclear ascertainment of the primary endpoint. This exclusion was pre-specified to ensure accurate and homogeneous phenotyping of AHF events. Inclusion criteria were: (1) age ≥18 years; (2) no history of atrial fibrillation; (3) admission for emergency PCI within 24 h of symptom onset; (4) complete demographic and clinical data available. Exclusion criteria included: (1) presence of malignant tumors; (2) non-obstructive coronary heart disease or primary cardiomyopathy; (3) evidence of clinical infection; (4) associated with autoimmune diseases; (5) severe liver and kidney dysfunction;(6)Patients with a previous diagnosis of heart failure (documented by previous medical records), heart failure-specific medication use, or echocardiographic findings consistent with chronic HF.

Acute Heart Failure Diagnostic Criteria and Event Adjudication: The diagnosis of acute heart failure was established using standardized criteria based on the 2021 ESC Guidelines for the diagnosis and treatment of acute and chronic heart failure and the 2022 AHA/ACC/HFSA Guideline for the Management of Heart Failure (13, 14). AHF was defined as the rapid onset or worsening of signs and symptoms of heart failure within the first 7 days following PCI, requiring urgent evaluation and treatment. All patients were systematically evaluated using the Killip classification system at admission and throughout hospitalization: Killip Class I (No clinical signs of heart failure), Killip Class II (Heart failure with rales and/or elevated jugular venous pressure), Killip Class III (Acute pulmonary edema), and Killip Class IV (Cardiogenic shock).

Diagnostic Criteria: (1) Clinical Manifestations: Presence of at least one of the following symptoms: dyspnea at rest or on minimal exertion, orthopnea, paroxysmal nocturnal dyspnea, or reduced exercise tolerance, combined with at least one of the following signs: elevated jugular venous pressure, pulmonary rales, peripheral edema, or third heart sound (S3 gallop). (2) Laboratory Evidence: Elevated NT-proBNP levels using age-adjusted thresholds: >450 ng/L (pg/mL) for patients <50 years, >900 ng/L for patients 50–75 years, and >1,800 ng/L for patients >75 years. For patients with renal insufficiency (estimated glomerular filtration rate <60 mL/min/1.73 m²), the threshold was adjusted to >1,200 ng/L regardless of age. (3) Imaging Evidence: Echocardiographic findings consistent with heart failure, including but not limited to: left ventricular ejection fraction ≤40% (HFrEF), evidence of diastolic dysfunction in patients with preserved ejection fraction (HFpEF), or signs of pulmonary congestion on chest radiography. Based on whether patients developed AHF after PCI, they were divided into the AHF group (*n* = 100) and the non-AHF group (*n* = 319). The study was approved by the Ethics Committee of the Maanshan People's Hospital, and the study protocol conformed to the principles of the Declaration of Helsinki.

### 2.2 Clinical data

Clinical information for all STEMI patients at hospital admission and during the initial evaluation period prior to PCI was collected, specifically including: age, gender, diabetes and hypertension status, smoking status, body mass index (BMI), baseline systolic and diastolic blood pressure upon admission, and heart rate upon admission. Baseline laboratory tests covered: neutrophils, lymphocytes, hemoglobin, platelets, total bilirubin, albumin, triglyceride (TG), total cholesterol (TC), low-density lipoprotein cholesterol (LDL-C), high density lipoprotein cholesterol (HDL-C), blood urea nitrogen, creatinine, uric acid, fasting blood glucose. Baseline LVEF was obtained from echocardiography performed during the initial hospitalization period. All predictor variables represent admission/baseline values obtained before or within the first few hours of PCI to enable early risk stratification. Interventional data included: Gensini score, door-to-balloon time (D-to-B time), site of infarction, and number of affected vessels. Hypertension and diabetes mellitus were identified according to established diagnostic criteria. Hypertension: history of diagnosis, current antihypertensive therapy, or three separate measurements of BP ≥ 140/90 mmHg (off acute stress; 1 mmHg = 0.133 kPa). Diabetes: documented diagnosis, antidiabetic medication use, or admission FPG ≥ 7.0 mmol/L, random glucose ≥ 11.1 mmol/L, or HbA1c ≥ 6.5%, per national and international guidelines.

### 2.3 Statistical analysis

Statistical analyses were conducted using SPSS 26.0 and R 4.2.0 software. Missing data rates for key predictive variables were minimal: systolic blood pressure (0.5%), neutrophil count (0.7%), total bilirubin (1.2%), urea nitrogen (0.9%), and LVEF (1.4%). Given the low overall missing data rate (<2%), listwise deletion (complete case analysis) was employed to maintain data integrity without introducing imputation bias. Multicollinearity assessment: Prior to multivariate analysis, we systematically evaluated multicollinearity among predictor variables using variance inflation factors (VIF) and tolerance statistics. All variables included in the final model demonstrated VIF values < 5.0 and tolerance values >0.2, indicating acceptable levels of multicollinearity. Model selection and regularization: We adhered strictly to the principle of at least 10 events per variable for multivariable modeling, limiting our final model to 5 predictors to ensure statistical robustness. No formal shrinkage or penalization methods were applied, as our conservative variable selection strategy provided sufficient protection against overfitting.

In the univariate analysis, normally distributed continuous data were presented as mean ± standard deviation, and intergroup comparisons were performed using independent samples t-test. Non-normally distributed data were presented as median and interquartile range [M(Q1, Q3)], with intergroup comparisons using the Mann–Whitney *U*-test. Categorical data were expressed as frequencies and proportions, and intergroup comparisons were conducted using the chi-square test.

For the multivariate analysis, ALL variables showing statistical significance (*P* < 0.05) in the univariate analysis were entered into the binary logistic regression model. These variables included: age, systolic blood pressure, diastolic blood pressure, neutrophil count, hemoglobin, total bilirubin, albumin, urea nitrogen, creatinine, fasting glucose, LVEF, and infarct location. Only variables maintaining statistical significance (*P* < 0.05) in the final multivariate model were retained as independent predictors and are presented in the results. We adhered to the principle of at least 10 events per variable for multivariable modeling. With 100 events of acute heart failure in our cohort of 419 patients, we limited the final number of predictors in the models to 6, thus ensuring statistical robustness and minimizing overfitting risk.

Binary logistic regression analysis was used to determine the independent factors influencing the occurrence of AHF after PCI in STEMI patients, and ROC curves were used to identify the threshold values and establish a predictive model. An omnibus test was performed for the overall evaluation of model coefficients, with a *P*-value of less than 0.05 indicating statistical significance. The Hosmer-Lemeshow test was used to assess the goodness of fit of the model; a *P*-value greater than 0.05 indicated good fit. At the same time, we performed ROC analysis and reclassification analysis to assess the potential value of prediction models in predicting AHF. The reclassification analysis included NRI and IDI. The Nomogram was built and validated based on the influencing factors of the prediction model. Additionally, clinical decision curve analysis was performed to evaluate the net benefit of the model for predicting AHF occurrence in STEMI patients following PCI. A *P*-value of less than 0.05 indicated that the differences were statistically significant.

## 3 Results

### 3.1 General conditions

A total of 419 STEMI patients who underwent PCI were included in this study. The results showed that 100 patients developed AHF during hospitalization, with an incidence rate of 23.86%. In the AHF group, there were 69 males and 31 females, with an average age of (65.68 ± 14.2) years; in the non-AHF group, there were 238 males and 81 females, with an average age of (60.77 ± 13.38) years.

### 3.2 General clinical data and univariate analysis

There were no significant differences between the AHF group and non-AHF group in terms of gender, BMI, diabetes, hypertension, smoking history, heart rate at admission, lymphocytes, platelets, triglycerides, total cholesterol, LDL-C, HDL-C, uric acid, Gensini score, D-to-B time, and the number of affected vessels (*P* > 0.05). However, there were significant differences between the two groups in terms of age, systolic and diastolic blood pressure at admission, neutrophils, hemoglobin, total bilirubin, albumin, blood urea nitrogen, creatinine, fasting blood glucose, LVEF, and infarct location (*P* < 0.05). These factors may be potential influencers of AHF occurrence in STEMI patients post-PCI (As shown in Tables 1, 2).

**Table 1:** Comparison of general clinical data between STEMI patients with AHF and without AHF.

Variables	AHF group (*n* = 100)	Non-AHF group (*n* = 319)	*t*/*χ²*/*z* Value	*P-*Value
Age (years)^b^	65.68 ± 14.20	60.77 ± 13.38	3.154	0.002*
Gender (Male, *n*%)	69 (69.00)	238 (74.60)	1.223	0.269
BMI, kg/m^2^	24.01 ± 4.00	24.43 ± 3.51	−0.519	0.605
Diabetes (*n*%)	32 (32.00)	81 (25.40)	1.688	0.194
Hypertension (*n*%)	53 (53.00)	161 (50.50)	0.195	0.659
Smoking (*n*%)	53 (53.00)	188 (58.90)	1.097	0.295
Heart rate (beats/min)^b^	78.00 (63.00, 90.00)	76.00 (68.00, 87.00)	−0.092	0.927
SBP (mmHg)^a^	109.93 ± 25.34	127.13 ± 22.64	−6.440	<0.001*
DBP (mmHg)^a^	66.81 ± 14.98	77.74 ± 15.64	−6.160	<0.001*
Neutrophils (×10^9^/L)^b^	8.87 (6.47, 11.66)	7.17 (5.20, 9.79)	4.020	<0.001*
Lymphocytes (×10^9^/L)^b^	1.30 (0.94, 2.51)	1.45 (1.06, 2.16)	−0.464	0.642
Hemoglobin (g/L)^a^	133.20 ± 19.47	138.31 ± 18.46	−2.381	0.018*
Platelets (×10^9^/L)^b^	195.00 (160.25, 237.50)	198.00 (156.00, 238.00)	0.128	0.898
Total bilirubin (umol/L)^b^	16.55 (11.60, 20.85)	18.10 (13.40, 24.70)	−2.669	<0.001*
Albumin(g/L)^a^	37.85 ± 4.08	39.57 ± 3.71	−3.936	<0.001*
Triglycerides (mmol/L)^b^	1.43 (0.98, 1.95)	1.50 (1.06, 2.15)	−1.201	0.230
Total cholesterol (mmol/L)^b^	4.30 (3.77, 4.92)	4.44 (3.84, 5.12)	−1.341	0.180
LDL-C (mmol/L)^b^	2.76 ± 0.80	2.76 ± 0.80	−1.727	0.085
HDL-C (mmol/L)^b^	1.10 ± 0.23	1.10 ± 0.26	−0.197	0.844
Urea (mmol/L)^b^	6.28 (5.10, 9.24)	5.14 (4.19, 6.38)	5.635	<0.001*
Creatinine (umol/L)^b^	77.00 (64.25, 122.15)	69.00 (58.50, 79.00)	4.169	<0.001*
Uric acid (umol/L)^b^	370.90 (301.63, 427.50)	346.00 (281.00, 415.00)	1.841	0.066
Glucose (mmol/L)^b^	7.27 (5.95, 10.13)	6.05 (5.31, 7.67)	4.241	<0.001*
LVEF^b^	58.00 (50.00, 61.75)	60.00 (55.00, 64.00)	−3.560	<0.001*
Gensini score^b^	56.00 (38.25, 94.00)	60.50 (42.00, 84.00)	0.212	0.832
D-to-B time	61.48 ± 8.20	60.95 ± 7.19	0.625	0.532
Infarct location (*n*, %)			4.255	0.039*
Anterior MI	44 (44.00)	178 (55.80)		
Others	56 (56.00)	141 (44.20)		
Number of diseased vessels (*n*, %)			2.711	0.100
1	72 (72.00)	201 (63.00)		
≥2	28(28.00)	118(37.00)		

SBP, systolic blood pressure; DBP, diastolic blood pressure; LDL-C, low-density lipoprotein cholesterol C; HDL-C, high-density lipoprotein cholesterol C; LVEF, left ventricular ejection fraction; BMI, body mass index.

* *P* < 0.05.

a Normally distributed data are expressed as mean ± standard deviation.

b Non-normally distributed data are expressed as median M (P25, P75).1 mmHg = 0.133 kPa.

**Table 2:** Univariate logistic regression analysis of AHF risk in STEMI patients.

Variables	Univariate Logistic regression analysis					
	β	S.E.	Wald	OR	95% CI	*P*
Age	0.027	0.009	9.552	1.027	1.010–1.045	0.002*
SBP	−0.033	0.006	34.289	0.967	0.957–0.978	<0.001*
DBP	−0.052	0.009	32.605	0.949	0.932–0.966	<0.001*
Neutrophils	0.132	0.031	18.414	1.142	1.075–1.213	<0.001*
Hemoglobin	−0.014	0.006	5.537	0.986	0.974–0.998	0.019*
Total bilirubin	−0.033	0.014	5.453	0.968	0.941–0.995	0.020*
Albumin	−0.118	0.031	14.432	0.889	0.837–0.945	<0.001*
Urea	0.310	0.051	37.153	1.363	1.234–1.506	<0.001*
Creatinine	0.022	0.004	28.975	1.022	1.014–1.030	<0.001*
Glucose	0.106	0.030	12.342	1.112	1.048–1.180	<0.001*
LVEF	−0.061	0.014	18.007	0.941	0.915–0.968	<0.001*
Infarct location	0.474	0.231	4.219	1.607	1.022–2.526	0.040*

SBP, systolic blood pressure; DBP, diastolic blood pressure; LVEF, left ventricular ejection fraction.

* *P* < 0.05.

### 3.3 Multivariate logistic analysis

**Figure 1:**
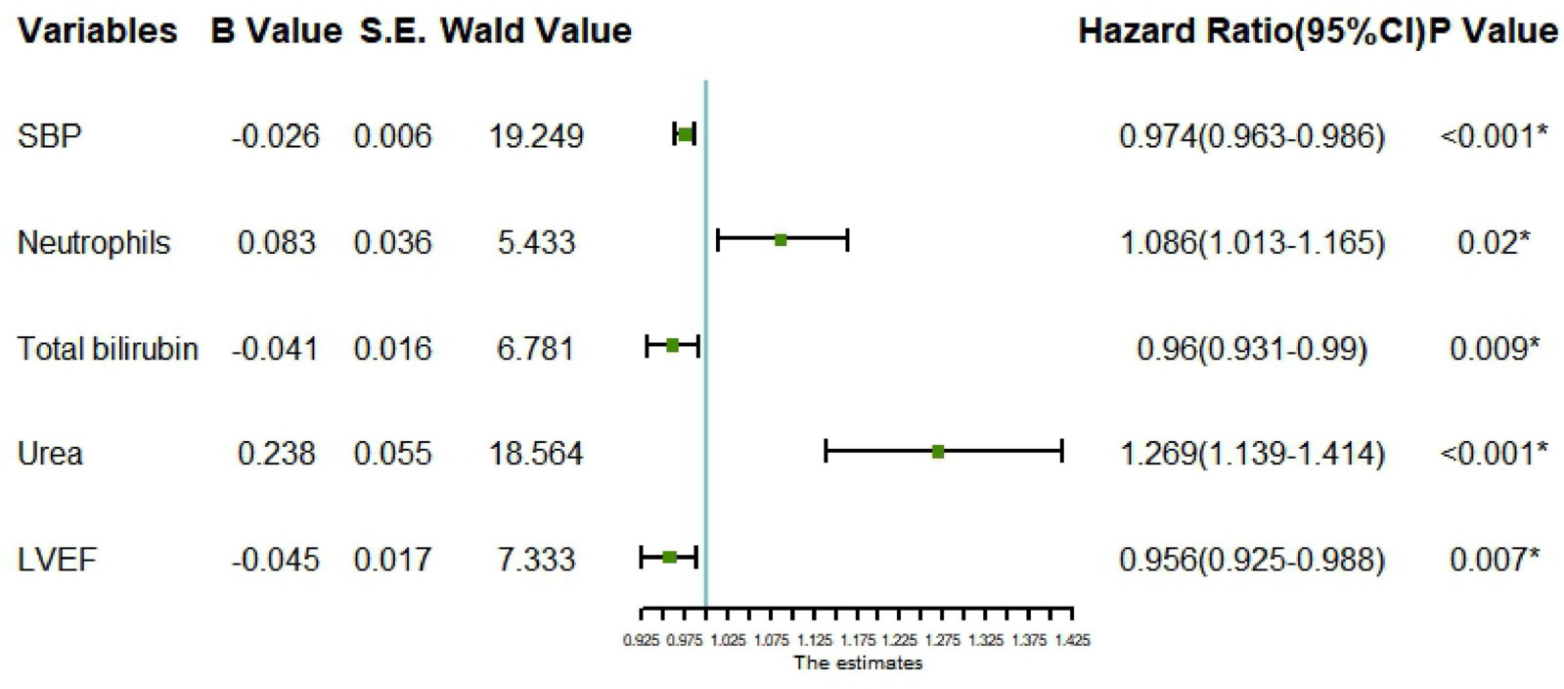
Forest plot for multivariate logistic regression analysis of risk of AHF in STEMI patients; SBP, systolic blood pressure; LVEF, left ventricular ejection fraction.

Multivariate logistic regression analysis results indicate that systolic blood pressure at admission, neutrophil levels, total bilirubin, urea nitrogen, and LVEF are independent factors influencing the occurrence of AHF after PCI in STEMI patients (*P* < 0.05), as detailed in Table 3 and Figure 1. Specifically, the effects are as follows: ① For each 1 mmHg increase in systolic blood pressure at admission, the probability of AHF occurrence decreases by 2.6%; ②For every 1 × 10^−9^/L increase in neutrophils, the probability of AHF occurrence increases by 8.6%; ③ For each 1 umol/L increase in total bilirubin, the probability of AHF occurrence decreases by 4%; ④ For each 1 mmol/L increase in urea nitrogen, the probability of AHF occurrence increases by 26.9%; ⑤ For each 1% increase in LVEF, the probability of AHF occurrence decreases by 4.4%.

**Table 3:** Multivariate logistic regression analysis of AHF risk in STEMI patients.

Variables	Multivariate Logistic regression analysis					
	β	S.E.	Wald	OR	95% CI	*P*
SBP	−0.026	0.006	19.249	0.974	0.963–0.986	<0.001*
Neutrophils	0.083	0.036	5.433	1.086	1.013–1.165	0.020*
Total bilirubin	−0.041	0.016	6.781	0.960	0.931–0.99	0.009*
Urea	0.238	0.055	18.564	1.269	1.139–1.414	<0.001*
LVEF	−0.045	0.017	7.333	0.956	0.925–0.988	0.007*

SBP, systolic blood pressure; LVEF, left ventricular ejection fraction.

* *P* < 0.05.

### 3.4 Model development and evaluation

The results of the multivariate logistic regression analysis indicated that admission systolic pressure, neutrophil count, total bilirubin, urea nitrogen, and LVEF are independent factors influencing the occurrence of AHF after PCI in STEMI patients. Through comprehensive analysis, a combined predictive model was established to enhance diagnostic accuracy, expressed as: Logit(P) = 3.084 − 0.026 × systolic pressure + 0.083 × neutrophil count − 0.041 × total bilirubin + 0.238 × urea nitrogen − 0.045 × LVEF. Note: The regression coefficients β for each covariate can be referenced in Table 3, and admission systolic pressure, neutrophil count, total bilirubin, urea nitrogen, and LVEF can be directly substituted into this formula for calculations.

**Figure 2:**
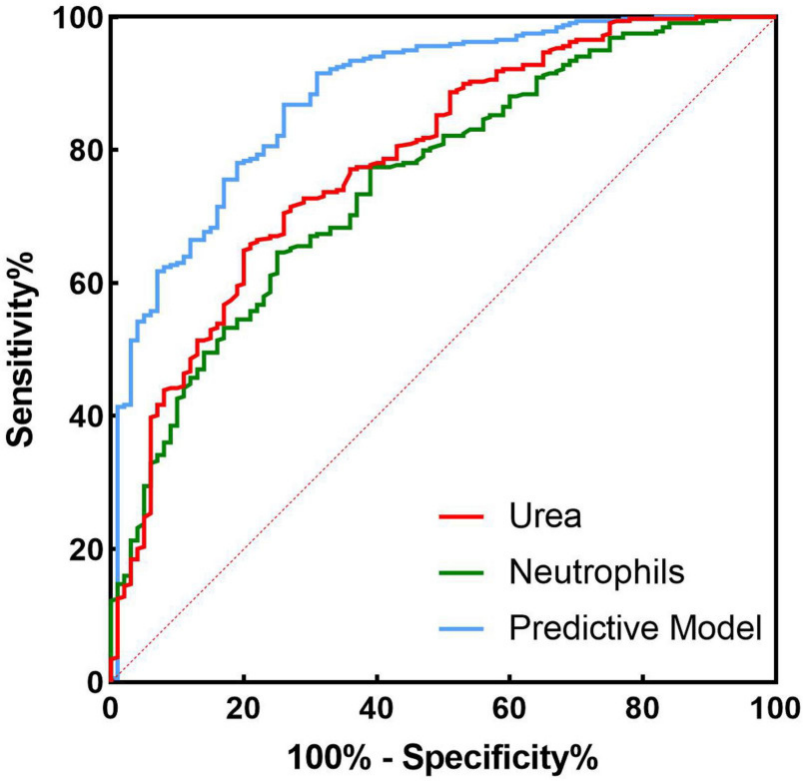
Receiver operating characteristic curve analysis of predictive models.

**Figure 3:**
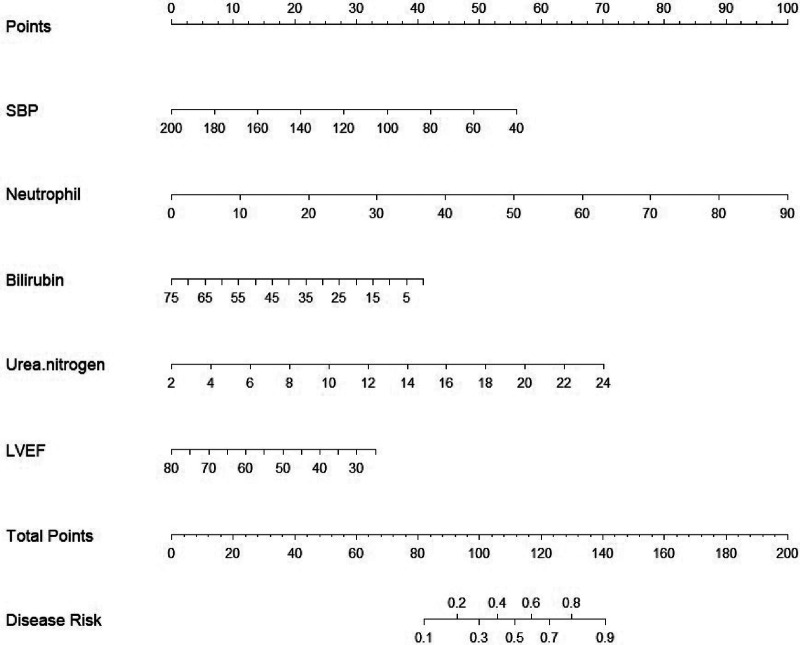
Nomogram for predicting AHF in STEMI patients.

**Figure 4:**
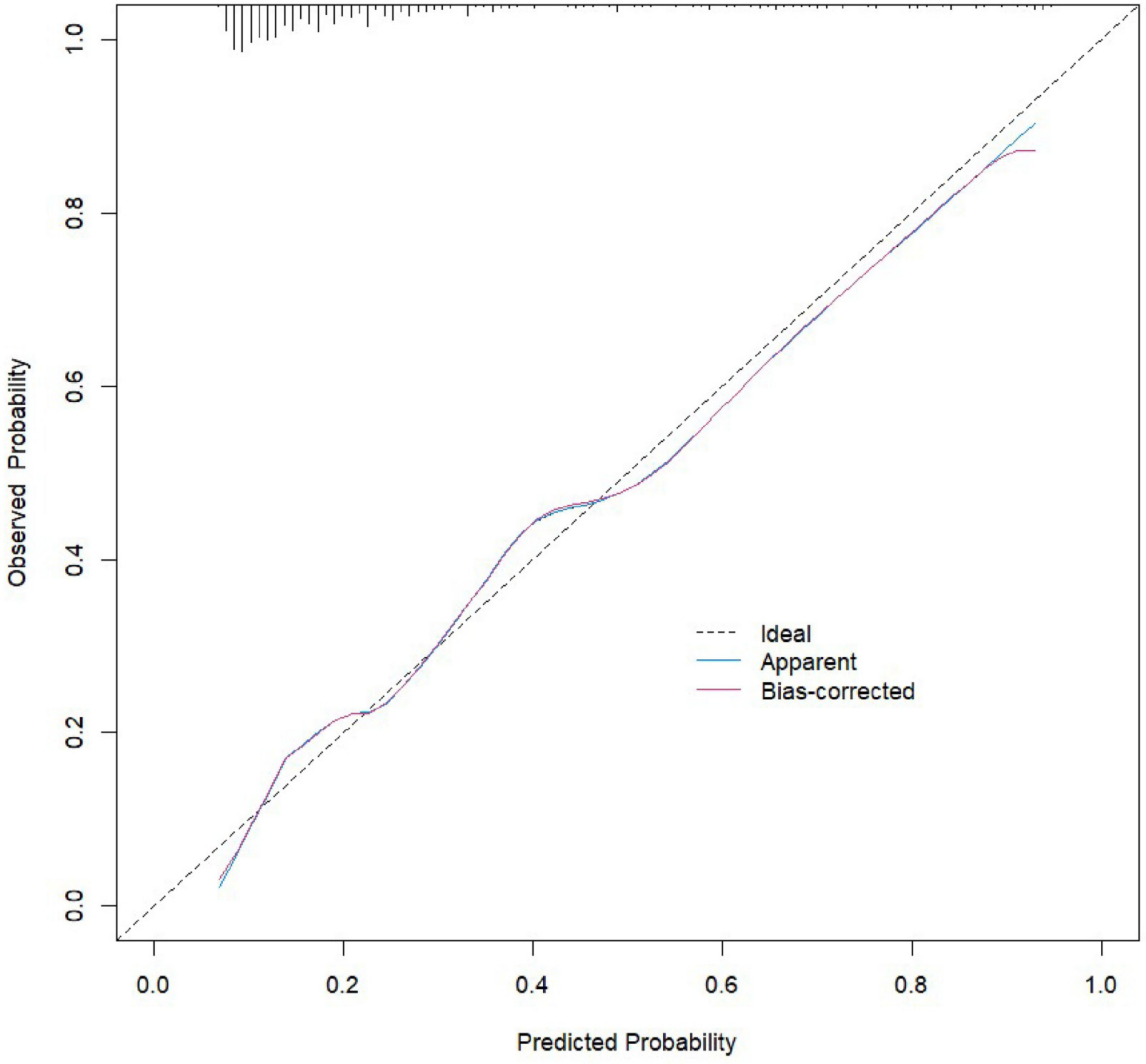
Calibration curve.

**Figure 5:**
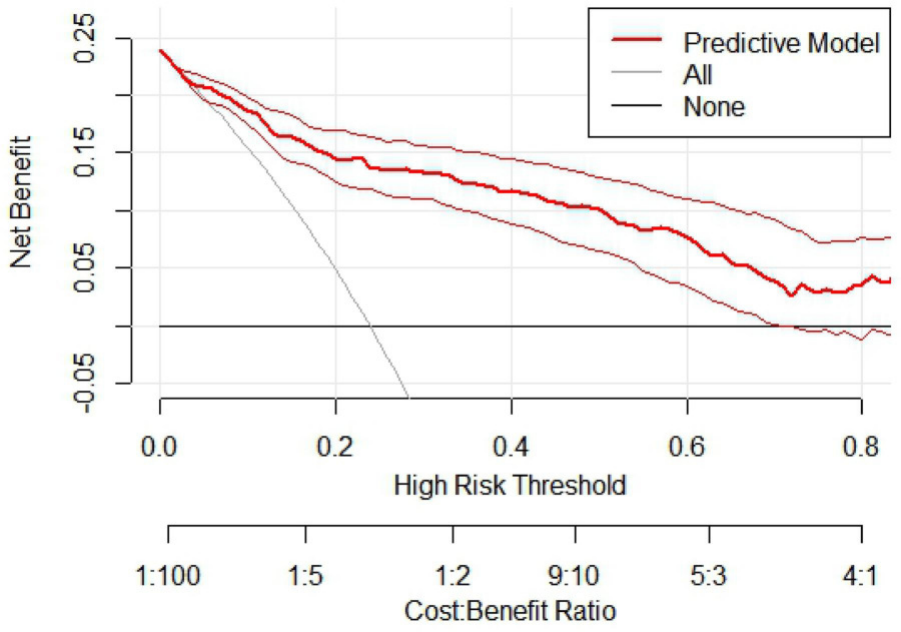
Clinical decision curve analysis for predictive models.

The results of the Omnibus test provided a comprehensive analysis of the model's coefficients, yielding *χ*² = 7.112, *P* = 0.008, indicating statistical significance. The Hosmer-Lemeshow test resulted in *χ*² = 6.551, *P* = 0.586 (>0.05), suggesting that the model fits well. The ROC curves and parameter estimates for the combined predictors and independent risk factors showed that the combined predictors significantly outperformed the individual risk factors in predicting AHF after PCI in STEMI patients, with the highest AUC value closest to the top left corner, as shown in Figure 2. The calculated Youden index indicated that the optimal cutoff value for the combined predictors was 0.447, with the best diagnostic threshold for Logit(P) at 0.231. When Logit(P) exceeds 0.231, it can predict the occurrence of AHF in STEMI patients after PCI. ROC and reclassification analyses were performed to evaluate the predictive model 's value in improving the AHF detection rate (Table 4 and Figure 2). In ROC analysis, the predictive model alone AUC was 0.883 (0.845–0.920). AUC for Grace Score was 0.715 (0.655–0.776); AUC for CAMI-STEMI was 0.717 (0.664–0.770). The AUC improved significantly with the addition of Grace Score to the predictive model (0.902 vs. 0.715, Delong test: *Z* = 0.235, *P* < 0.0001). On reclassification analysis, continuous NRI was 0.7472 (0.5378 to 0.9565) and IDI was 0.2455 (0.1735 to 0.3176). AUC was also significantly improved by adding CAMI-STEMI to the predictive model (0.909 vs. 0.717, Delong test: *Z* = 0.4930, *P* < 0.0001). Continuous NRI of 0.7245 (0.5122–0.9369) and IDI of 0.2101 (0.1306–0.2896) confirmed that the prediction model significantly improved the detection rate of AHF. Nomogram established by the predictive model, Figure 3. The results of H-L goodness-of-fit test of Nomogram for predicting AHFE in STEMI patients showed *χ*² = 9.7905, *P* = 0.28, and the model calibration curve was close to the ideal model with strong calibration. As shown in Figure 4, the internal validation C-index was 0.818 (95% CI: 0.78–0.87), suggesting that the model had good calibration; clinical decision curve analysis showed that the model provided a net clinical benefit to patients between 0.1 and 0.99 at a threshold probability, as shown in Figure 5. Beyond the Hosmer-Lemeshow test (*χ*² = 6.551, *P* = 0.586), we performed additional calibration analyses to assess agreement between predicted and observed probabilities. Calibration slope was 0.856 (95% CI: 0.721–0.991), indicating good agreement between predicted and observed risks, with values close to 1.0 representing ideal calibration. The calibration intercept was 0.089 (95% CI: −0.023–0.201), demonstrating minimal systematic bias in risk prediction, as values close to 0 indicate optimal calibration. These metrics, combined with the visual calibration curve (Figure 4) showing close alignment with the ideal diagonal line, confirm excellent model calibration across the full range of predicted probabilities.

**Table 4:** ROC and reclassification analysis for predictive model to refine the detection of AHF.

Model	AUC (95% CI)	*P* value	*P* for comparison	NRI (Continuous)	*P* value	IDI	*P* value
Grace Score	0.715 (0.655–0.776)	<0.0001	–	–	–	–	–
CAMI-STEMI	0.717 (0.664–0.770)	<0.0001	–	–	–	–	–
Predictive Model	0.883 (0.845–0.920)	<0.0001	–	–	–	–	–
Predictive Model + Grace Score	0.902 (0.864–0.939)	<0.0001^a^	0.00019	0.7472 (0.5378–0.9565)	<0.0001	0.2455 (0.1735–0.3176)	<0.0001
Predictive Model + CAMI-STEMI	0.909 (0.887–0.940)	<0.0001^b^	<0.0001	0.7245 (0.5122–0.9369)	<0.0001	0.2101 (0.1306–0.2896)	<0.0001

NRI, net reclassification index; IDI, integrated discrimination improvement.

a Delong test: *Z* = 0.235, *P* < 0.0001.

b Delong test: *Z* = 0.4930, *P* < 0.0001.

## 4 Discussion

STEMI represents one of the most severe cardiovascular emergencies, typically caused by acute complete coronary occlusion. While PCI has become the standard treatment for rapidly restoring myocardial blood flow and improving survival rates (15, 16), post-procedural complications remain critical determinants of long-term outcomes despite significant advances in PCI techniques (17). Cardiac function recovery post-PCI is closely related to reperfusion timeliness, myocardial ischemia extent, and post-procedural management quality (4, 7). Therefore, individualized management strategies targeting high-risk patients are essential, including cardiac function monitoring, optimized pharmacological treatment, and lifestyle interventions to reduce complications.

AHF represents a common and serious complication among STEMI patients, occurring in 23.86% of cases in this study (11). Its development is influenced by multiple factors including patient age, gender, underlying conditions, myocardial ischemia severity, reperfusion effectiveness, and post-procedural management quality (18–20). AHF occurrence significantly increases hospital length of stay and mortality risk, adversely affecting long-term prognosis (8, 21). Among affected patients, ventricular remodeling and cardiac dysfunction rates increase substantially, further exacerbating clinical outcomes and impacting quality of life (22). Early identification of high-risk patients and implementation of appropriate interventions are crucial for improving outcomes.

Our analysis of 419 STEMI patients who underwent PCI revealed that 100 patients (23.86%) developed AHF during hospitalization. The AHF group demonstrated older age (65.68 ± 14.2 vs. 60.77 ± 13.38 years, with 69 males), consistent with previous research indicating high AHF incidence in STEMI patients and close associations with age, sex, and comorbidities (3, 4). Older patients are more susceptible to post-infarction heart failure, likely related to physiological cardiac aging and increased comorbidity burden (7).

Univariate analysis revealed no significant differences between AHF and non-AHF groups regarding gender, BMI, diabetes, hypertension, and smoking history (*P* > 0.05). However, significant differences emerged in age, systolic blood pressure, diastolic blood pressure, neutrophil count, and hemoglobin levels (*P* < 0.05). These findings indicate that age and hemodynamic parameters are important predictive factors, consistent with literature identifying age, heart failure, and underlying diseases as independent AHF predictors (8, 23). Patients with existing heart failure demonstrate significantly increased AHF risk following acute myocardial infarction, emphasizing the importance of careful assessment in STEMI patients (24–26).

Multivariate logistic regression analysis confirmed that admission systolic blood pressure, neutrophil count, total bilirubin, blood urea nitrogen, and LVEF are independent factors influencing post-PCI AHF occurrence in STEMI patients. Specifically, each 1 mmHg increase in admission systolic blood pressure was associated with a 2.6% decrease in AHF probability, while each 1 × 10^−9^/L increase in neutrophil count corresponded to an 8.6% increase in AHF probability. These results align with existing literature highlighting the importance of cardiac function and biochemical indicators in AHF development (27–29). Elevated neutrophil levels reflect cardiac inflammatory responses that may increase AHF risk by promoting myocardial injury (30).

Based on these findings, we established a multifactorial logistic regression prediction model incorporating admission systolic blood pressure, neutrophil count, total bilirubin, blood urea nitrogen, and LVEF as independent AHF predictors (31, 32). This comprehensive analysis enables more accurate AHF prediction, providing a robust foundation for clinical decision-making. The model's excellent fit and high predictive accuracy demonstrate significant practical value in clinical practice (33, 34).

While some core predictors such as LVEF and systolic blood pressure overlap with traditional risk models, our study provides incremental value through several key innovations. By integrating clinically available biomarkers including neutrophil count, total bilirubin, and urea nitrogen, our model captures additional pathophysiological dimensions of inflammation, hepatic function, and renal function that complement existing risk assessment tools. The model's specific focus on the early post-PCI period in STEMI patients offers clinicians a practical tool for prompt risk stratification and intervention. The promising AUC, NRI, and IDI values suggest improved predictive performance compared to GRACE and CAMI-STEMI scores in our single-center cohort, though external validation remains essential to confirm these findings and establish broader applicability. Future research should continue exploring additional potential biomarkers to further refine the AHF prediction model and assess its applicability across different populations. Through continuous optimization, clinicians will be better equipped to identify high-risk patients and develop personalized treatment plans, ultimately improving patient outcomes and quality of life.

An important observational finding in our study is the negative correlation between total bilirubin levels and AHF risk (*β* = −0.041, *P* = 0.009), where each 1 μmol/L increase in total bilirubin was associated with a 4% decrease in AHF probability. While our cross-sectional study design cannot establish causality, this association may be consistent with emerging evidence suggesting potential cardioprotective properties of bilirubin through multiple biological pathways. Previous experimental studies have suggested that bilirubin may function as an endogenous antioxidant, potentially scavenging reactive oxygen species (ROS) and reducing oxidative stress, processes that have been implicated in myocardial injury progression and subsequent heart failure development (35). Laboratory studies have also indicated that bilirubin may possess anti-inflammatory properties by potentially inhibiting pro-inflammatory cytokines and reducing endothelial activation, mechanisms that could theoretically contribute to myocardial protection during the critical post-PCI period (36). Some experimental evidence has suggested that physiological levels of bilirubin might protect endothelial function by preventing endothelial cell apoptosis and maintaining nitric oxide bioavailability, potentially improving microcirculation and reducing myocardial dysfunction following PCI (37). Several observational studies have reported inverse associations between serum bilirubin levels and cardiovascular events, with patients having higher bilirubin levels within the normal range showing lower risks of heart failure and cardiovascular mortality (38). However, it is crucial to emphasize that these associations do not imply causation, and our findings represent an observational relationship that requires further investigation through prospective studies and mechanistic research to establish any potential causal relationship between bilirubin levels and cardiac outcomes.

It is crucial to emphasize that this study represents an exploratory, hypothesis-generating investigation rather than a definitive clinical tool ready for immediate implementation. While our model demonstrated promising discriminative performance (AUC 0.883) and good calibration in this single-center cohort, these findings should be interpreted with appropriate caution. The model requires rigorous external validation in independent, multicenter populations across diverse healthcare systems, patient demographics, and clinical practices before it can be considered for routine clinical use. Such validation studies are essential to confirm the model's generalizability, assess its performance across different populations, and establish its true clinical utility in real-world settings.

The negative correlation between admission systolic blood pressure and AHF occurrence (*β* = −0.026, *P* < 0.001) reflects the critical importance of hemodynamic reserve in post-PCI outcomes. Higher systolic blood pressure within physiological ranges indicates preserved cardiac contractility and vascular tone, maintaining adequate perfusion pressure gradients for optimal coronary perfusion and end-organ function (39). Conversely, lower admission blood pressure often signifies compromised cardiac output, reduced stroke volume, or early hemodynamic decompensation, predisposing to acute heart failure development. This protective effect of adequate systolic pressure has been consistently observed in acute coronary syndrome populations, where hypotension correlates with increased mortality and heart failure complications (40). Notably, this relationship applies to blood pressure levels within normal to mildly elevated ranges, excluding hypertensive crisis scenarios where extremely elevated pressures may be detrimental.

The negative correlation between LVEF and AHF risk (*β* = −0.045, *P* = 0.007) represents the most clinically intuitive relationship, where each 1% LVEF increase corresponded to a 4.4% decrease in AHF probability. As a direct quantitative measure of left ventricular systolic function and contractile capacity, LVEF serves as a fundamental heart failure risk determinant (41). Preserved LVEF indicates better adaptation to hemodynamic stress from acute myocardial infarction and PCI procedures, maintaining adequate cardiac output despite injury. This well-established relationship between reduced LVEF and increased heart failure risk makes LVEF a cornerstone parameter in heart failure classification and risk stratification (42), reinforcing the importance of early echocardiographic assessment for identifying high-risk post-PCI patients (43).

This model specifically targets STEMI patients without previous heart failure, predicting *de novo* acute heart failure occurrence after PCI. Performance in established heart failure patients or for predicting chronic heart failure worsening was not evaluated. While point-based scoring systems offer rapid bedside triage value, we prioritized preserving full predictive performance using the continuous logit(P) formula, supplemented by information systems and graphical nomogram tools for practical bedside assessment. Future studies with larger, diverse populations may consider simplified integer-based scoring.

Our predictive model achieved superior performance (AUC 0.883) compared to established scoring systems through innovative variable selection and pathophysiological comprehensiveness. The GRACE score incorporates eight variables (age, heart rate, systolic blood pressure, creatinine, Killip class, cardiac arrest at admission, ST-segment deviation, and elevated cardiac enzymes) for general acute coronary syndrome risk stratification. The CAMI-STEMI score includes five variables (age, Killip class, systolic blood pressure, heart rate, and creatinine) targeting STEMI mortality prediction rather than specific complications. In contrast, our model integrates five carefully selected variables capturing distinct pathophysiological pathways: systolic blood pressure (hemodynamic status), neutrophil count (inflammatory response), total bilirubin (hepatic function and hemolysis), urea nitrogen (renal function), and LVEF (cardiac contractility). This combination enables comprehensive assessment of multiple organ systems involved in post-PCI acute heart failure development.

Several mechanistic advantages explain our model's superior performance (AUC 0.883 vs. GRACE 0.715 and CAMI-STEMI 0.717): Enhanced inflammatory assessment through neutrophil count captures acute inflammatory responses following myocardial infarction and PCI, which traditional scores inadequately address. Hepatic function integration via total bilirubin identifies early cardiac dysfunction indicators preceding clinical heart failure manifestations, enabling earlier risk identification than clinical classification systems like Killip class. Quantitative cardiac function assessment using continuous LVEF measurements provides more precise evaluation than categorical Killip classifications employed by GRACE and CAMI-STEMI scores. Post-PCI specificity addresses unique pathophysiological changes during myocardial reperfusion, whereas traditional scores target broader acute coronary syndrome populations. The significant improvements in continuous NRI (0.7472 for GRACE, 0.7245 for CAMI-STEMI) and IDI (0.2455 and 0.2101 respectively) demonstrate clinically meaningful patient reclassification, identifying high-risk individuals missed by traditional systems while appropriately reclassifying low-risk patients.

Rather than replacing established scoring systems, our model offers complementary value. The GRACE score (AUC 0.715) remains the gold standard for general acute coronary syndrome risk stratification with comprehensive clinical variables including age, Killip class, and cardiac arrest history (44). The CAMI-STEMI score (AUC 0.717) maintains validated clinical utility for STEMI mortality prediction. Our model adds incremental predictive value by incorporating biochemical markers reflecting distinct pathophysiological pathways not fully captured by clinical classification systems. This represents incremental enhancement rather than paradigm shift in risk prediction. The biological plausibility of neutrophil count (inflammatory response), total bilirubin (antioxidant capacity), and urea nitrogen (renal function) as AHF predictors is well-established (45, 46). Our contribution lies in systematic integration within a STEMI-specific, post-PCI context, providing complementary information to existing clinical risk factors. The promising performance metrics (continuous NRI 0.7472, IDI 0.2455) indicate potential clinical utility, but external validation across diverse populations remains essential before clinical implementation. Single-center retrospective analysis limits generalizability, requiring confirmation across different healthcare systems, patient populations, and clinical practices to establish robust evidence for routine use.

An important methodological limitation of our study relates to the variable selection strategy employed. Our use of univariate significance testing (*P* < 0.05) as the criterion for inclusion in multivariate modeling, while widely accepted in clinical research, carries inherent limitations that merit acknowledgment. This approach may inadvertently exclude clinically meaningful predictors that fail to achieve statistical significance in univariate analysis due to confounding, interaction effects, or insufficient power. Conversely, it may retain variables that achieve significance by chance, particularly given multiple testing without formal adjustment.

Modern statistical approaches such as penalized regression methods (LASSO, Ridge regression, or Elastic Net) offer potential advantages by simultaneously performing variable selection and coefficient shrinkage, thereby reducing overfitting risk while potentially identifying optimal predictor combinations independent of traditional *P*-value thresholds. These methods can capture complex predictor relationships and may be particularly valuable in scenarios with moderate sample sizes relative to the number of potential predictors. Additionally, knowledge-driven variable selection based on established pathophysiological mechanisms, regardless of univariate significance, represents another valid approach that could complement or replace statistical significance-based selection.

However, our conservative approach was deliberately chosen to balance statistical rigor with clinical interpretability, ensuring model stability within our sample size constraints while maintaining biological plausibility of included predictors. The resulting model demonstrated robust performance with good discrimination (AUC 0.883), excellent calibration, and meaningful clinical reclassification ability. Nevertheless, future research would benefit from comparative studies evaluating different variable selection strategies, including penalized regression methods and knowledge-driven approaches, to optimize both predictive accuracy and clinical utility in diverse populations.

This study has several limitations. Firstly, and most importantly, this is an exploratory, single-center retrospective study, which fundamentally limits the generalizability and external validity of our findings. The retrospective design may introduce selection and information bias, while the single-center nature means our results may not be representative of patient populations from other regions, hospitals, or healthcare systems with different clinical practices, patient demographics, or treatment protocols. This model should be considered hypothesis-generating and exploratory until external validation in independent, multicenter populations confirms its performance and clinical utility. Secondly, the research data comes from the cardiology department of Maanshan People's Hospital, which may limit the external validity of the results and not represent patient populations from other regions or hospitals. Thirdly, although our inclusion and exclusion criteria and standardized treatment protocols effectively controlled for major confounding factors, including standardization of postoperative medications, unmeasured confounders may still exist, such as patients’ lifestyle factors, medication compliance variations, and genetic factors. Fourthly, while the model performed well in this study, its applicability and generalizability need to be verified in different populations and clinical settings to ensure its effectiveness for broad application. Fifthly, the study primarily focuses on the occurrence of postoperative AHF and lacks follow-up data on patients’ long-term prognosis, making it impossible to assess the model's impact on long-term management and outcomes. Additionally, while clinical and laboratory data were collected during hospitalization, the precise timing of covariate measurements relative to PCI procedures was not systematically standardized across all patients, which could potentially affect the model's predictive accuracy. Finally, although the sample size was modest, we carefully controlled the number of covariates. However, external validation in a larger multicenter cohort is required. In summary, this research has important clinical significance and scientific value in establishing a prediction model for AHF after PCI in STEMI patients, but it also has drawbacks such as a retrospective design, single-center data source, and the omission of other potential influencing factors. Future research should aim to conduct broader validation and supplementation based on these findings.

In summary, this study clarifies the independent predictive value of various clinical indicators in the occurrence of AHF and presents an exploratory model that shows promise for risk stratification. These findings provide important reference points for clinicians in managing STEMI patients and generate hypotheses for future validation studies. Before clinical implementation, this model requires rigorous external validation in independent, multicenter populations to confirm its performance, assess generalizability, and establish clinical utility. Furthermore, future studies should continue to explore other potential biomarkers and clinical characteristics to further refine predictive models for AHF and comprehensively assess their applicability and effectiveness across diverse populations and healthcare settings.

## Data Availability

The raw data supporting the conclusions of this article will be made available by the authors, without undue reservation.
